# Effects of Spirulina Supplementation on C‐Reactive Protein (CRP): A Systematic Review and Dose–Response Meta‐Analysis

**DOI:** 10.1002/fsn3.70196

**Published:** 2025-05-05

**Authors:** Mostafa Shahraki Jazinaki, Mohammad Rashidmayvan, Pegah Rahbarinejad, Mohammad Reza Shadmand Foumani Moghadam, Naseh Pahlavani

**Affiliations:** ^1^ Department of Nutrition, Faculty of Medicine Mashhad University of Medical Sciences Mashhad Iran; ^2^ Department of Nutrition, Food Sciences and Clinical Biochemistry, School of Medicine, Social Determinants of Health Research Center Gonabad University of Medical Sciences Gonabad Iran; ^3^ Service of Clinical Nutrition and Dietitian, Emam Reza Hospital Mashhad University of Medical Sciences Mashhad Iran; ^4^ Health Sciences Research Center Torbat Heydariyeh University of Medical Sciences Torbat Heydariyeh Iran; ^5^ Social Determinants of Health Research Center Torbat Heydariyeh University of Medical Sciences Torbat Heydariyeh Iran

**Keywords:** cardiovascular diseases, C‐reactive protein, inflammation, spirulina

## Abstract

The preceding research has produced varied results concerning the impact of Spirulina supplementation on C‐reactive protein (CRP) levels, considered one of the primary risk factors associated with inflammation in chronic conditions. We aimed to understand the potential relationship between Spirulina supplementation and human CRP modulation by performing a meta‐analysis. A comprehensive search was conducted up to February 2024 on prominent medical bibliographic databases, including Web of Science, Scopus, and PubMed, to identify relevant studies. The overall effect size was calculated using a random‐effects model that was proposed by DerSimonian and Laird. The pooled effect size was expressed as weighted mean differences (WMD) with 95% confidence intervals (CI). Furthermore, the heterogeneity between the included studies was evaluated using Cochran's *Q* test and the *I* ^2^ statistic. Our meta‐analysis included 7 trials with 283 subjects and 10 effect sizes. Spirulina supplementation significantly reduced serum CRP levels compared to the control group (WMD: −0.55 mg/L; 95% CI: −0.90 to −0.21; *p* = 0.002). However, heterogeneity among the studies was high (*I* ^2^ = 86.7%, *p* < 0.001). Furthermore, no significant linear and non‐linear relationship between supplementation features (dosage and duration) and changes in CRP levels was detected. The results of the current systematic review and meta‐analysis suggest that the intake of Spirulina can cause significant decreases in CRP levels. However, more extensive and well‐executed studies are still needed to draw definitive conclusions regarding this effectiveness.

## Graphical abstract

Our meta‐analysis, which combined data from seven trials (with 10 arms), revealed that Spirulina supplementation led to a significant decrease in C‐reactive protein levels compared to control groups.

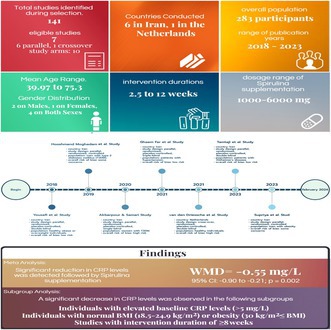

## 1 Introduction

C‐reactive protein (CRP) is recognized as an acute‐phase protein produced through IL‐6‐dependent hepatic biosynthesis. It indicates significantly elevated levels in response to injuries, infections, and inflammatory conditions (Sproston and Ashworth 2018). CRP is a valuable clinical marker of inflammation, and an elevated CRP level is recognized as a robust independent predictor of cardiovascular disease (CVD). It plays a crucial role in the pathophysiology of CVD, highlighting its significance in assessing and understanding cardiovascular health (Ridker et al. 2002). In addition, individuals with CRP levels above 3 mg/L have an increased risk of coronary heart disease (CHD) (Kushner 1990) and type 2 diabetes (Soinio et al. 2006), which is higher than others with lower levels. Furthermore, studies have demonstrated the association between CRP level and the prognosis of atherosclerotic disease, congestive heart failure (CHF), atrial fibrillation, myocarditis, aortic valve disease, and heart transplantation (Osman et al. 2006). Recent investigations have suggested that specific nutraceuticals can positively impact biochemical factors, such as high‐sensitivity CRP (hs‐CRP) (Zaplatic et al. 2019; Hamaguchi et al. 2010). In this context, Spirulina is a suitable complementary therapy to help reduce inflammation (Yousefi et al. 2018). The Spirulina platensis (*Arthrospira platensis*) is a filamentous cyanobacterium that has recently gained significant popularity as a nutritional supplement, primarily due to its remarkable biological properties, including immunomodulatory, antioxidant, and anti‐inflammatory effects (Wu et al. 2016). Among 35 various species of Spirulina, Spirulina platensis is the most widely used in the diet and food industry (Yukesh Kannah et al. 2021). Furthermore, the anti‐inflammatory effects attributed to Spirulina are widely acknowledged as among its most significant advantages. Ongoing research endeavors continue to delve into its potential applications in managing inflammatory conditions while concurrently promoting overall health. Notably, Spirulina exhibits a pronounced capacity for inflammation attenuation, as evidenced by its capability to effectively diminish concentrations of interleukin‐2, adiponectin, and tumor necrosis factor‐α (Lee et al. 2008). Distinguished by its vibrant blue‐green hue, Spirulina has earned the moniker “the best food for the future.” This microalga is classified within the cyanobacterial family (Karkos et al. 2008; Gumbo and Nesamvuni 2017). It boasts abundant bioactive constituents, encompassing protein (55% to 70%), carbohydrates (comprising 10 to 14%), and lipids (approximately 4 to 9%). Additional components include fiber, ash, water, diverse minerals, vitamins, γ‐linolenic acid, chlorophyll, carotenoids, and the notable phycocyanin (Brito et al. 2020). Previous studies have evaluated the effects of Spirulina on conditions such as Alzheimer's disease (AD) (Tamtaji et al. 2023), healthy individuals with overweight or obesity (Yousefi et al. 2018), and hypertension (Ghaem Far et al. 2021). For example, a study conducted by Tamtaji et al. demonstrated that the consumption of Spirulina for 12 weeks among patients with Alzheimer's disease resulted in enhanced cognitive function, improved glucose homeostasis parameters, and decreased levels of hs‐CRP (Tamtaji et al. 2023). In another study by Yousei et al., a significant reduction in Hs‐CRP levels was observed in participants who received Spirulina supplementation compared to the control group (Yousefi et al. 2018). It is worth highlighting that these studies exhibited variations in terms of population, baseline inflammatory characteristics, intervention duration, and Spirulina dosage. Until now, no systematic review study has comprehensively assessed the impact of Spirulina on CRP levels. As a result, our current study stands as the inaugural systematic review and meta‐analysis aimed at addressing this knowledge gap and evaluating Spirulina's influence on CRP levels.

## 2 Material and Methods

Every stage of this systematic review and meta‐analysis adhered closely to Preferred Reporting Items for Systematic Reviews and Meta‐Analyses (PRISMA) framework (Moher et al. 2009).

The procedural framework for conducting this systematic review has been registered in the PROSPERO database, and the registration code assigned is: CRD42023446673.

### 2.1 Search Strategy

A comprehensive systematic search encompassing databases such as Web of Science, Scopus, and PubMed was carried out until February 2024. The objective of the search was to find studies investigating the impact of Spirulina supplementation on CRP levels. This search had no restrictions in language or time.

The employed search strategy encompassed both MeSH and non‐MeSH keywords, which were as follows: (“Spirulina” OR “Arthrospira”) AND (“inflammation” OR “c‐reactive protein” OR “CRP” OR “Hs‐CRP” OR “High sensitivity C‐reactive protein”) AND (*“*intervention*”* OR “randomized” OR “Randomized controlled trial” OR “RCT”). The reference lists of the related studies and Google Scholar were checked to avoid missing eligible studies.

### 2.2 Study Selection

The findings from the initial search underwent screening by two researchers, (M.S.J.) and (P.R.), following the eligibility criteria. Any differences in opinion were thoroughly discussed until a consensus was reached.

Inclusion criteria included:

- Human studies
- Trials conducted on adults (≥ 18 years)
- Studies in which the intervention group received Spirulina
- Studies that reported serum CRP changes during the intervention
- Studies with appropriate control groups

Furthermore, combined treatment with Spirulina, not having a control group, not reporting serum CRP changes, and non‐human interventional studies such as review articles, animal studies, short communication studies, or letters to the editor were deemed exclusion criteria.

### 2.3 Data Extraction

Two researchers (M.S.J.) and (M.R.) independently extracted information related to the objectives of this systematic review. The following information was extracted: name of the first author, year of publication, country, population characteristics (health status and mean baseline BMI and age of participants in each group), dose and duration of intervention with Spirulina, type of intervention in the control group, and mean changes and standard deviation of serum CRP levels during the study.

### 2.4 Quality Assessment

The assessment of study quality for the included studies was conducted independently by (P.R.) and (M.S.J.) using the Cochrane Risk of Bias tool (ROB 2) (Higgins and Green 2009). The evaluation covered five aspects of bias risk: bias arising from the randomization process, bias due to deviations from intended interventions, bias due to missing outcome data, bias in measurement of the outcome, and bias in selection of the reported result. Any discrepancies were resolved through consultation with a third researcher (N.P.). The risk of biases in each part was classified as low risk, some concerns, and high risk.

### 2.5 Statistical Analysis

The overall effect size was calculated using the mean changes and standard deviations (SDs) for CRP levels in each included studies. The overall effect size was express as Weighted Mean Diffrences (WMD) with 95% confidence interval (95% CI) (DerSimonian and Laird 1986). If SDs were not reported in a study, they were calculated based on medians, interquartile ranges, and standard error (SE) using the protocol of Hozo et al. (Hozo et al. 2005). If mean changes of CRP levels were not reported directly, it was calculated by subtracting measures in baseline from the end of intervenion. Also, SD changes during the intervention were calculated using the following formula (Borenstein et al. 2021): Change SD = square root [(SDbaseline)^2^ + (SDfinal)^2^−(2 × *R* × SDbaseline × SDfinal)]. Heterogeneity among studies was evaluated using Cochran's *Q* test and the *I*‐square (*I* ^2^) statistic (Higgins et al. 2003). *I* ^2^ > 40% or *p*‐value < 0.05 were interpreted as a significant heterogeneity among studies (Higgins and Thompson 2002). Publication bias in this meta‐analysis was measured by the Egger’s regression test (Egger's test) and visual interpretation of the funnel plots (Egger et al. 1997). A sensitivity analysis was performed to check the effect of omitting each of the included studies on the pooled overall effect size (Tobias 1999). Meta‐regression and dose–response analysis were performed to investigate the linear and non‐linear relationship between the intervention with Spirulina (dose and duration) and CRP level changes, respectively (Mitchell 2012). The following predetermined criteria were used to do subgroup analysis in order to identify the potential source of heterogeneity: Country of origin (None‐Iran vs. Iran), mean age of volunteers (N.R, < 50, and ≥ 50), baseline CRP (≤ 5, and > 5 mg/L), trial duration (< 8 VS ≥ 8 weeks), Spirulina dosage (≤ 1500, and > 1500 mg/day), sexes (both, males, and females), baseline BMI, and health status. All of the analyses conducted in this study were performed using Stata software version 17 (Stata Corp, College Station, TX). In all analyses, *p*‐values < 0.05 were interpreted as statistical significance.

## 3 Results

### 3.1 Study Selection

**FIGURE 1:**
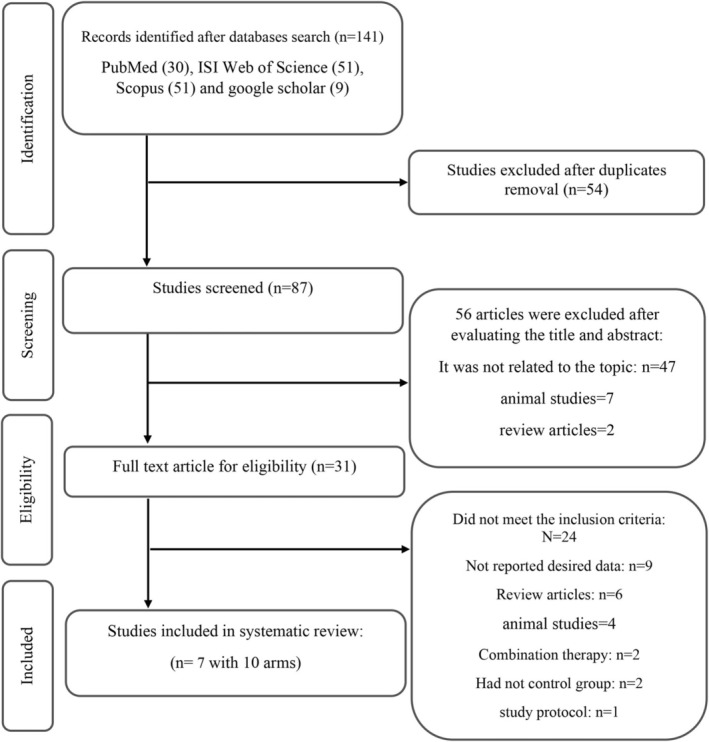
Flow chart for selecting eligible studies for inclusion in this systematic review.

Of the initial 141 studies identified in the first search, 54 were identified as duplicates and removed. Screening the remaining studies based on their title and abstract led to the exclusion of 56 unrelevant studies. The full text of the 31 remaining articles was carefully reviewed. After that, 24 studies were excluded due to combination therapy (*n* = 2), animal studies (*n* = 4), not reporting desired data (*n* = 9), not having a control group (*n* = 2), and review articles (*n* = 6), study protocols (*n* = 1). Finally, 7 eligible studies (comprising 10 arms) with 283 participants were included in this systematic review (Figure 1) (Yousefi et al. 2018; Tamtaji et al. 2023; Ghaem Far et al. 2021; Akbarpour and Samari 2020; van den Driessche et al. 2021; Hooshmand Moghadam et al. 2022; Supriya et al. 2023).

### 3.2 Findings of Systematic Review

The characteristics of all the studies included in this review are shown in Table 1 (Yousefi et al. 2018; Tamtaji et al. 2023; Ghaem Far et al. 2021; Akbarpour and Samari 2020; van den Driessche et al. 2021; Hooshmand Moghadam et al. 2022; Supriya et al. 2023). The included studies were published between 2018 (Yousefi et al. 2018) and 2023 (Tamtaji et al. 2023; Supriya et al. 2023). The mean age of the participants was in the range of 39.9 (Yousefi et al. 2018) to 75.3 years (Tamtaji et al. 2023), while the age of participants was not reported in 2 included trials (Hooshmand Moghadam et al. 2022; Supriya et al. 2023). Among the included studies, 6 had a parallel design (Yousefi et al. 2018; Tamtaji et al. 2023; Ghaem Far et al. 2021; Akbarpour and Samari 2020; Hooshmand Moghadam et al. 2022; Supriya et al. 2023), and 1 had a crossover design (van den Driessche et al. 2021). Furthermore, 2 studies were conducted on males (Hooshmand Moghadam et al. 2022; Supriya et al. 2023), 1 on females (Akbarpour and Samari 2020), and 4 on both sexes (Yousefi et al. 2018; Tamtaji et al. 2023; Ghaem Far et al. 2021; van den Driessche et al. 2021). In 3 studies, the mean serum CRP levels at baseline were ≤ 5 mg/dL (Tamtaji et al. 2023; Ghaem Far et al. 2021; Akbarpour and Samari 2020), and in 3 studies were > 5 mg/L (Yousefi et al. 2018; Hooshmand Moghadam et al. 2022; Supriya et al. 2023). However, the mean serum CRP levels at baseline were not reported in one included study (van den Driessche et al. 2021). In the included trials, 6 studies were conducted in Iran (Yousefi et al. 2018; Tamtaji et al. 2023; Ghaem Far et al. 2021; Akbarpour and Samari 2020; Hooshmand Moghadam et al. 2022; Supriya et al. 2023), and one was conducted in the Netherlands (van den Driessche et al. 2021). The Spirulina supplementation dosage in different studies was between 1000 (Tamtaji et al. 2023; Hooshmand Moghadam et al. 2022) and 6000 mg/day (Supriya et al. 2023), and the duration of the intervention ranged from 2.5 (van den Driessche et al. 2021) to 12 weeks (Yousefi et al. 2018; Tamtaji et al. 2023; Supriya et al. 2023). The population of participants included healthy subjects (Yousefi et al. 2018; van den Driessche et al. 2021) and people with diabetes (Akbarpour and Samari 2020; Hooshmand Moghadam et al. 2022), hypertension (Ghaem Far et al. 2021), and Alzheimer (Tamtaji et al. 2023). However, in one study, the health state of the subjects was not reported (Supriya et al. 2023).

**TABLE 1:** Characteristic of included studies in meta‐analysis.

Studies Country	Study design	Participant	Sample size and gender type	Sample size		Trial duration (week)	Means age		Means BMI		Intervention	
				IG	CG		IG	CG	IG	CG	Spirulina dose (mg/d)	Control group
Yousefi et al. (2018) Iran	Parallel, R, PC, DB	Healthy obese or overweight individuals	38 B	19	19	12	40.16 ± 10.8	39.79 ± 8.26	32.67 ± 4.49	32.99 ± 4.29	2 g/d Spirulina Platensis (two tablets in the morning and two tablets in the evening, after meals) + low‐calorie diet	Placebo + low‐calorie diet
Akbarpour and Samari (2020) (a) Iran	Parallel, R, PC, SB (semi‐experimental study)	Women with T2DM	20 F	10	10	6	46.28 ± 6.21	47.16 ± 7.44	31.01 ± 5.18	28.91 ± 2.62	1.5 g/d Spirulina (one capsule before each main meal)	Placebo
Akbarpour and Samari (2020) (b) Iran	Parallel, R, PC, SB (semi‐experimental study)	Women with T2DM	20 F	10	10	6	50.87 ± 8.60	49.57 ± 5.76	29.77 ± 3.19	29.14 ± 2.34	1.5 g/d Spirulina (one capsule before each main meal) + AT	Placebo + AT
Ghaem Far et al. (2021) Iran	Parallel, R, PC, TB	Patients with hypertension	41 B	22	19	8	51.27 ± 1.30	50.21 ± 1.36	29.75 ± 0.98	29.78 ± 1.61	2 g/d Spirulina (Arthrospira platensis in sachet form)	Placebo
van den Driessche et al. (2021) Netherlands	Cross‐over, R, PC, DB	Healthy individuals	35 B	35	35	2.5	40.2 ± 19.6	40.2 ± 19.6	24.7 ± 2.7	24.7 ± 2.7	4.8 g/d Spirulina (4 capsules directly after breakfast, lunch, and dinner)	Placebo
Hooshmand Moghadam et al. (2022) (a) Iran	Parallel, R, C (semi‐experimental study)	Men with T2DM	16 M	8	8	8	35–55	35–55	NR	NR	1 g/d Spirulina	Without supplementation
Hooshmand Moghadam et al. (2022) (b) Iran	Parallel, R, C (semi‐experimental study)	Men with T2DM	16 M	8	8	8	35–55	35–55	NR	NR	1 g/d Spirulina + 3 AT/w	3 AT/w
Tamtaji et al. (2023) Iran	Parallel, R, PC, DB	Alzheimer's disease	53 B	27	26	12	73.8 ± 9.9	76.9 ± 5.4	21.9 ± 2.2	23.3 ± 3.0	1 g/d Spirulina (Arthrospira platensis)	Placebo
Supriya et al. (2023) (a) Iran	Parallel, R, C	Men with Obesity	22 M	11	11	12	NR	NR	33.31 ± 0.62	32.77 ± 1.18	6 g/d Spirulina (one capsule in the morning and the other in the evening)	Placebo
Supriya et al. (2023) (b) Iran	Parallel, R, C	Men with Obesity	22 M	11	11	12	NR	NR	33.00 ± 1.00	33.01 ± 0.76	6 g/d Spirulina (one capsule in the morning and the other in the evening) + 3 high‐intensity interval training/week	Placebo + 3 high‐intensity interval training/week

Abbreviations: AT, aerobic training; BMI, body mass index; CG, control group; CO, controlled; DB, double‐blinded; F, female; IG, intervention group; M, male; NR, not reported; PC, placebo‐controlled; RA, randomized; SB, single‐blinded; T2DM, type 2 diabetes; NR, not reported.

**FIGURE 2:**
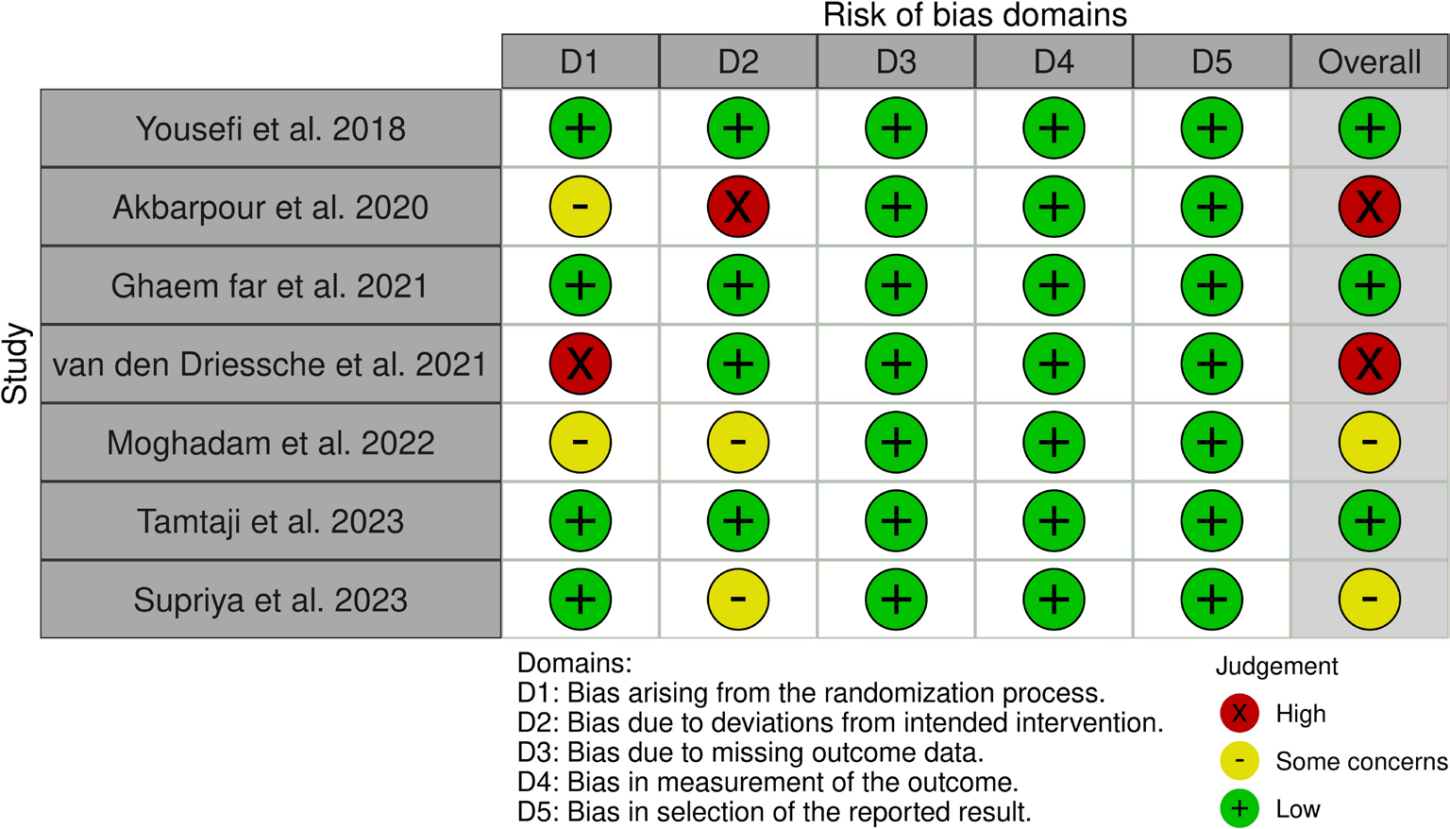
Risk of bias assessment plot.

In the risk of bias assessment, which was conducted based on the risk of bias assessment tool introduced by Cochrane (Higgins and Green 2009), the general risk of bias was identified as low in 3 (Yousefi et al. 2018; Tamtaji et al. 2023; Ghaem Far et al. 2021), with some concerns in 2 (Hooshmand Moghadam et al. 2022; Supriya et al. 2023), and high in 2 included trials (Akbarpour and Samari 2020; van den Driessche et al. 2021). Details of the risk of bias assessment are presented in Figure 2.

### 3.3 Meta‐Analysis

#### 3.3.1 Effect of Spirulina Supplementation on CRP Levels

**FIGURE 3:**
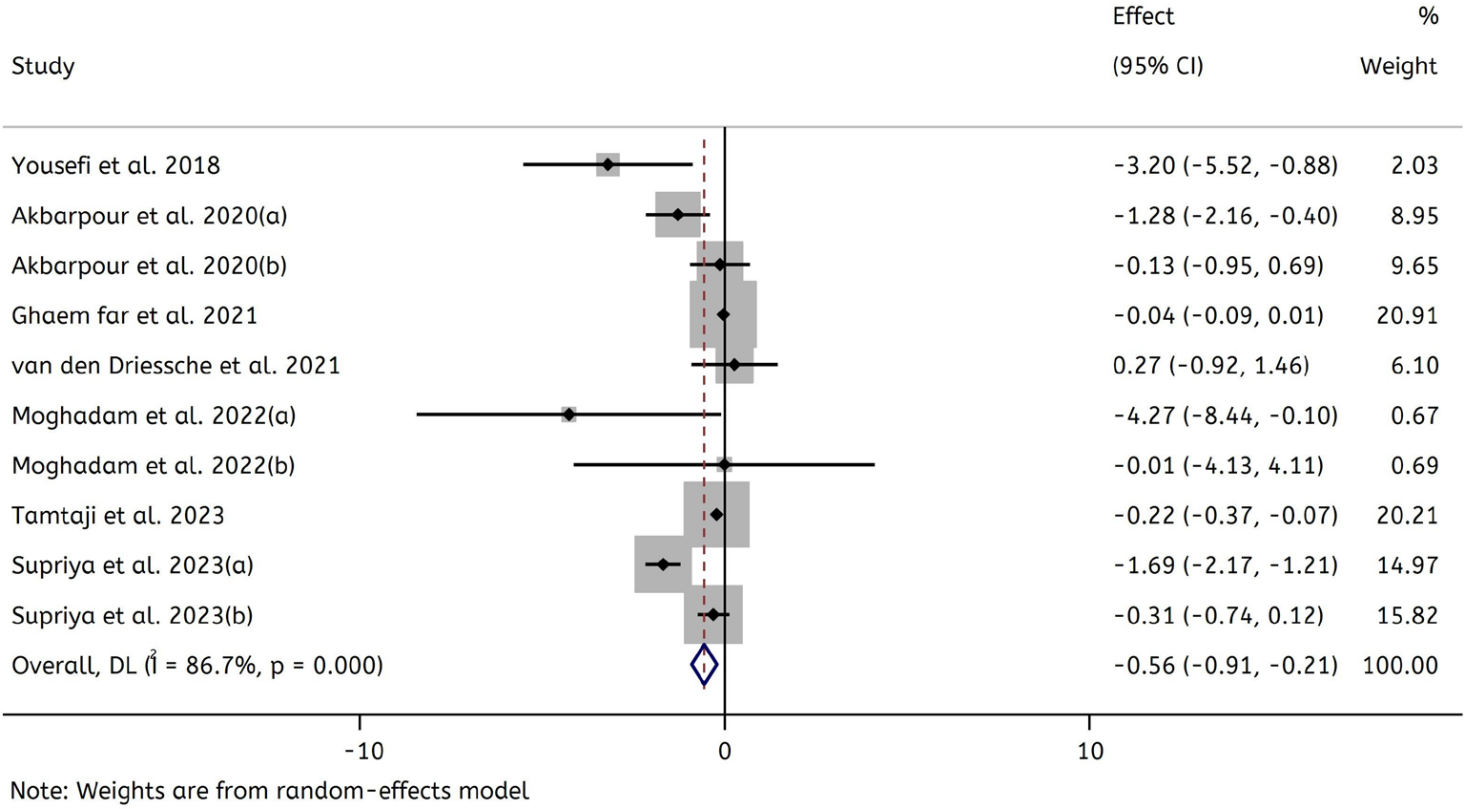
Forest plot representing weighted mean difference and 95% confidence intervals (CIs) for the effect of Spirulina supplementation on CRP levels (mg/L).

Combining 10 effect sizes showed that Spirulina supplementation led to a significant decrease in serum CRP level compared to the control groups (WMD: −0.55 mg/L; 95% CI: −0.90 to −0.21; *p* = 0.002). Heterogeneity among the pooled studies was significantly high (*I* ^2^ = 86.7%, *p* < 0.001) (Figure 3).

### 3.4 Subgroup Analysis

Subgroup analysis showed that Spirulina supplementation significantly reduced the CRP serum levels in the studies with an intervention duration of ≥ 8 weeks or trials conducted on the Iranian population, individuals with obesity or normal BMI, as well as participants with elevated CRP levels at baseline (> 5 mg/L) (Table 2).

**TABLE 2:** Subgroup analyses of Spirulina supplementation on serum CRP level.

	NO	WMD (95% CI)	*p*	Heterogeneity		
				*P* heterogeneity	*I* ^2^	*P* between sub‐groups
Subgroup analyses of Spirulina supplementation on serum CRP						
Overall effect	10	−0.55 (−0.90, −0.21)	**0.002**	**< 0.001**	86.7%	
Country						
Iran	9	−0.61 (−0.97, −0.25)	**0.001**	**< 0.001**	88.1%	0.16
None‐Iran	1	0. 27 (−0.91, 1.45)	0.65	—	—	
Age						
50 >	3	−1. 15 (−2.71, 0.41)	0.14	**0.01**	75.7%	0.11
50 ≤	3	−0. 11 (−0. 26, 0. 03)	0.14	0.08	59.3%	
NR	4	−1. 16 (−2.41, 0.08)	0.06	**< 0.001**	85.0%	
Baseline CRP (mg/L)						
*x* ≤ 5	4	−0. 19 (−0. 42, 0. 03)	0.10	**0.007**	75.5%	0.07
*x* > 5	5	−1. 48 (−2.67, −0.29)	**0.01**	**< 0.001**	83.0%	
NR	1	0. 27 (−0.91, 1.45)	0.65	—	—	
Trial duration (week)						
*x* < 8	3	−0.42 (−1.32, 0.47)	0.35	0.06	63.0%	0.73
*X* ≥ 8	7	−0. 59 (−0. 99, −0. 19)	**0.003**	**< 0.001**	90.1%	
Intervention dose (mg/day)						
*x* ≤ 1500	5	−0. 54 (−1. 19, 0. 10)	0.09	0.05	55.9%	0.75
*X* > 1500	5	−0.71 (−1.50, 0.08)	0.08	**< 0.001**	92.5%	
Health status						
Healthy	3	−1.31 (−4.70, 2.07)	0.44	**0.009**	85.3%	0.72
T2DM	3	−0. 88 (−2.01, 0.24)	0.12	0.09	83.3%	
Other health conditions	4	−0.48 (−0.86, −0.10)	**0.01**	**< 0.001**	93.9%	
Gender						
Both sexes	4	−0.14 (−0. 38, 0. 10)	0.26	**0.007**	75.4%	0.19
Male	4	−1. 16 (−2.41, 0.08)	0.06	**< 0.001**	85.0%	
Female	2	−0. 69 (−1.82, 0.43)	0.22	0.06	71.4%	
Baseline BMI (kg/m^2^)						
Normal (18.5–24.9)	2	−0.21 (−0.36, −0.06)	**0.005**	0.42	0.0%	0.24
Overweight (25–29.9)	3	−0. 39 (−1.07, 0.29)	0.26	**0.02**	73.8%	
Obese (> 30)	3	−1. 39 (−2. 67, −0. 11)	**0.03**	**< 0.001**	90.5%	
NR	2	−2.12 (−6.30, 2.04)	0.31	0.15	50.7%	

*Note:* Bold indicates statistical significance value (*p* < 0.05).

Abbreviations: BMI, body mass index; CI, confidence interval; CRP, C‐reactive protein; NR, not reported; T2DM, Type 2 diabetes; WMD, weighted mean difference.

### 3.5 Meta‐Regression

**FIGURE 4:**
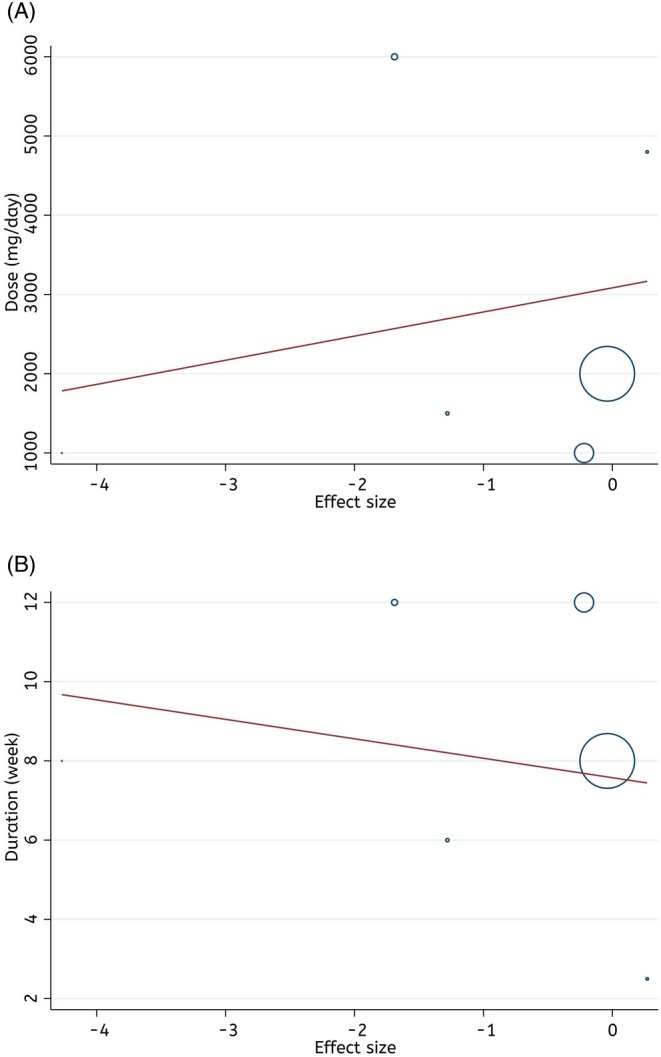
Linear dose–response relations between features of Spirulina supplementation (A) dose (mg/day), and (B) duration (weeks) and absolute mean differences in CRP (mg/L).

Meta‐regression analysis showed no linear relationship between the Spirulina supplementation features and changes in CRP levels (dose: coefficients = 304.67, *P*_linearity_ = 0.64, and duration: coefficients = −0.49, *P*_linearity_ = 0.69). Therefore, the dose and duration of supplementation with Spirulina were not sources of heterogeneity (Figure 4A,B).

### 3.6 Dose–Response Analysis

**FIGURE 5:**
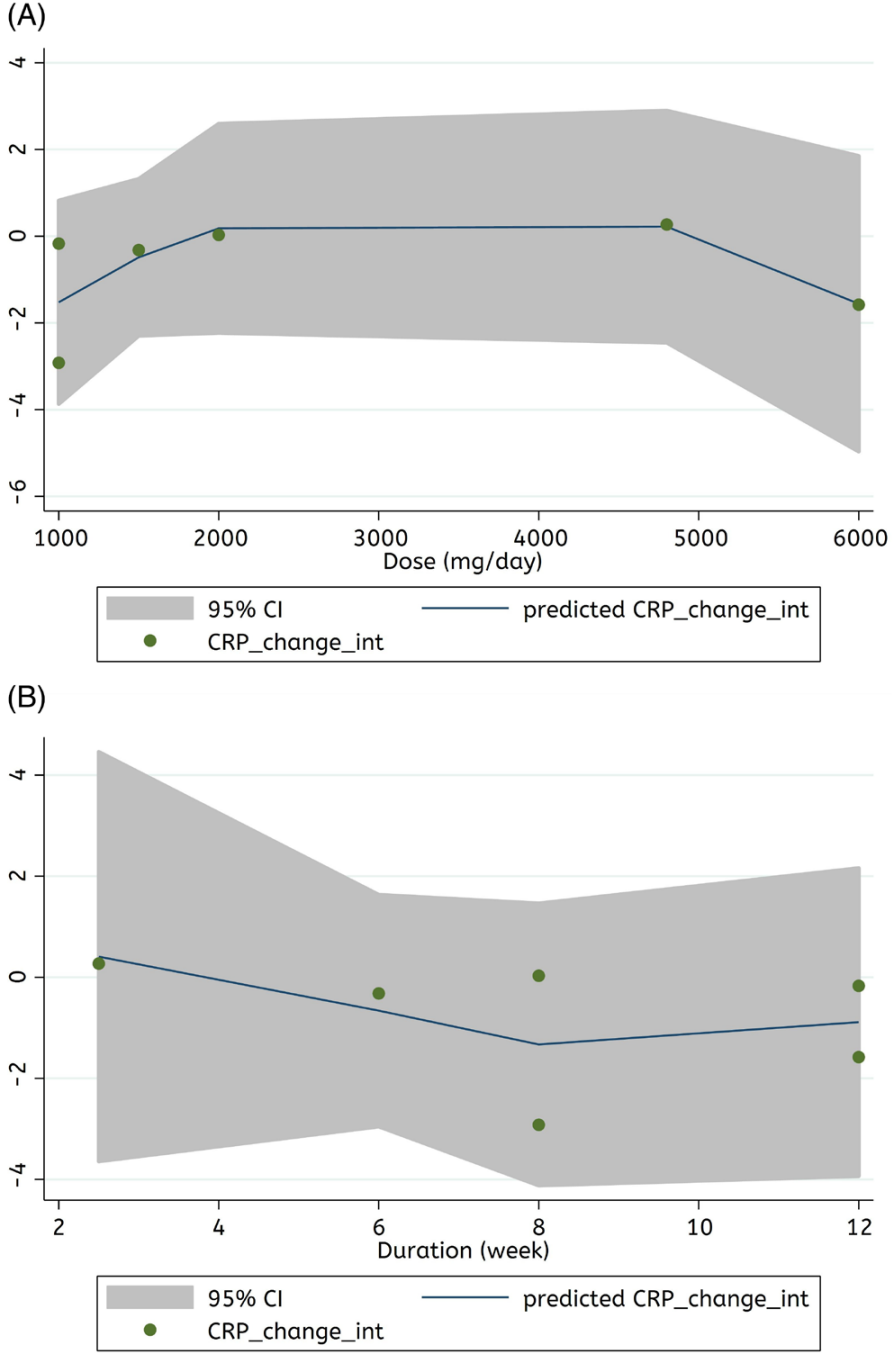
Non‐linear dose–response relations between features of Spirulina supplementation (A) dose (mg/day), and (B) duration (weeks) and absolute mean differences in CRP (mg/L).

Fractional polynomial modeling showed that there is no significant non‐linear relationship between the Spirulina supplementation characteristics and changes in CRP levels (dose: coefficients = −0.02, *P*_non−linearity_ = 0.20, and duration: coefficients = 0.007, *P*_non−linearity_ = 0.39) (Figure 5A,B).

### 3.7 Publication Bias and Sensitivity Analysis

**FIGURE 6:**
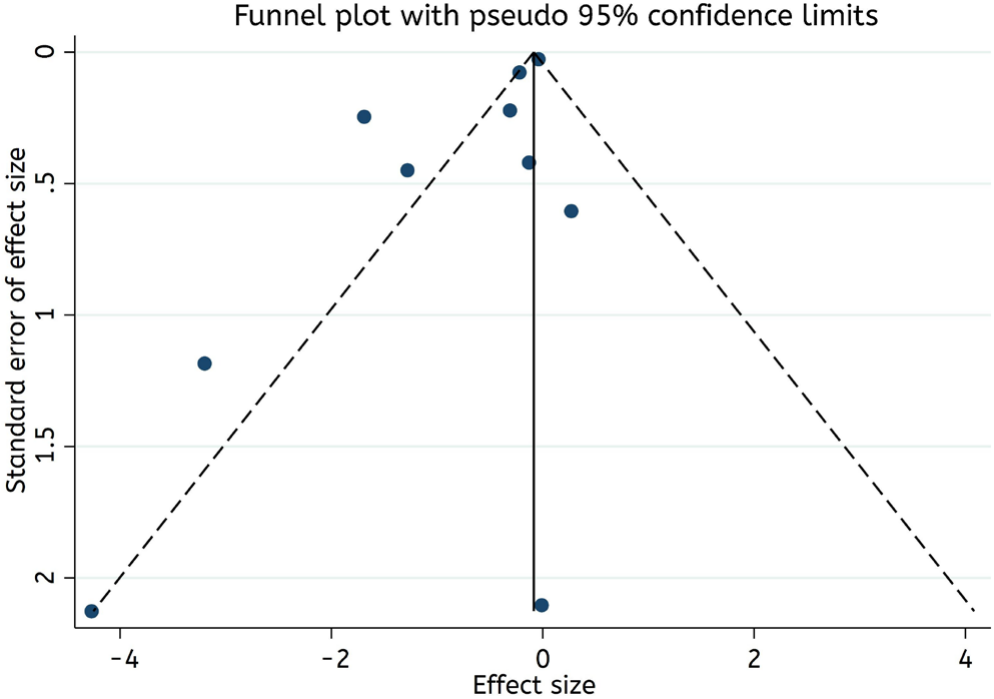
Funnel plots for the effect of Spirulina supplementation on CRP (mg/L).

Egger's regression test and the visual interpretation of the funnel plot showed a significant publication bias among studies investigating the effect of Spirulina supplementation on serum CRP levels (*p* = 0.04) (Figure 6). Furthermore, sensitivity analysis revealed that omitting any of the included studies did not lead to a significant change in the overall effect size.

## 4 Discussion

**FIGURE 7:**
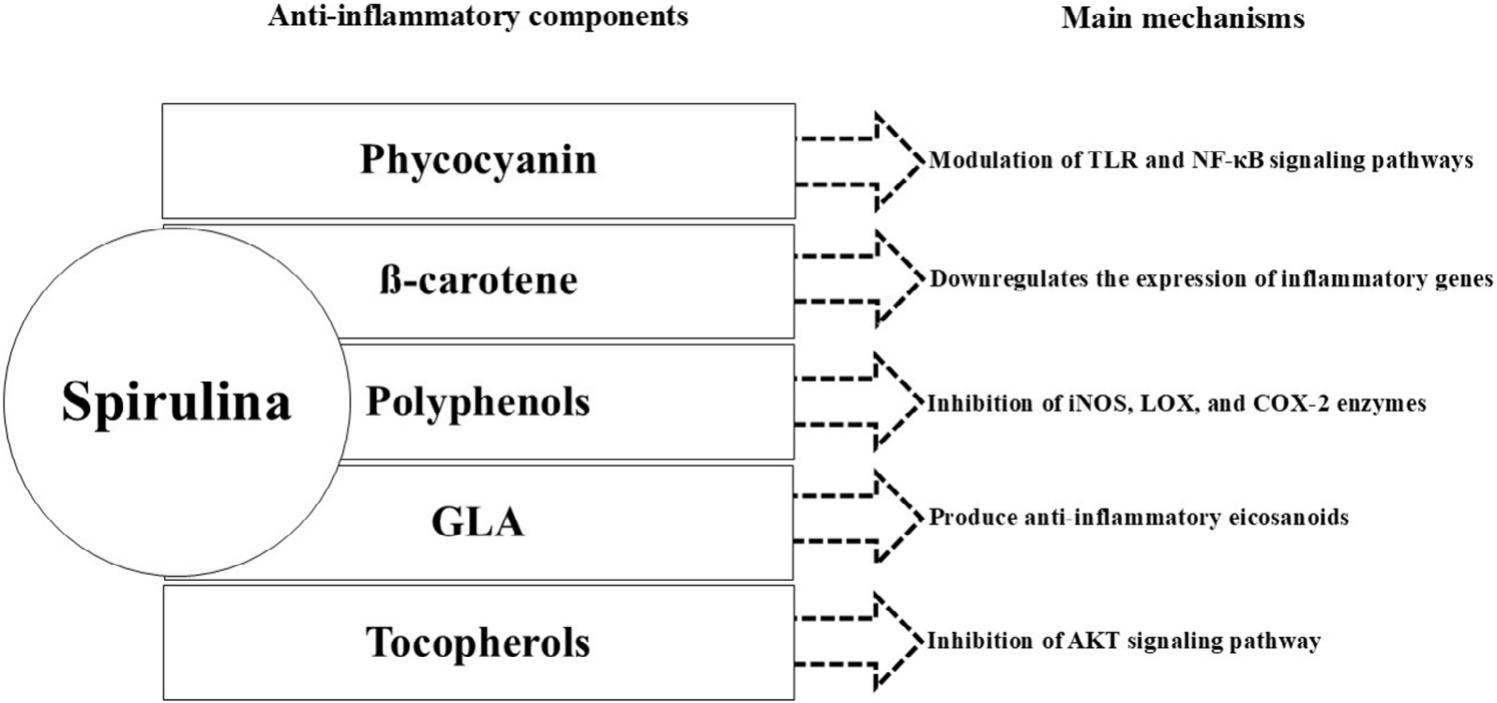
Possible anti‐inflammatory mechanisms of spirulina components.

In the current systematic review and meta‐analysis, we aimed to assess the role of Spirulina supplementation on the levels of CRP levels. After screening, we included 7 eligible studies for this systematic review (involving 283 participants). Our results indicated that Spirulina supplementation significantly reduced serum CRP levels compared to the control group (WMD: −0.55 mg/L; 95% CI: −0.90 to −0.21; *p* = 0.002). However, there was significant heterogeneity among the included trials (*I* ^2^ = 86.7%, *p* < 0.001). Furthermore, no significant linear or non‐linear relationship was observed between changes in CRP levels and Spirulina supplementation dose and duration. Notably, some of the key constituents in Spirulina align with components found in anti‐inflammatory dietary patterns, such as omega‐3 fatty acids and polyphenols (Hassan and El‐Gharib 2015). Furthermore, the observed positive impact of Spirulina on inflammatory status may be attributed to the presence of phycocyanin, ß‐carotene, gamma linolenic acid (GLA), tocopherols, and polyphenols, which seem to be the most potent anti‐inflammatory and antioxidant components within Spirulina (Wu et al. 2016, 2023; Serban et al. 2016; Priyanka et al. 2023; Grammas et al. 2004; Yahfoufi et al. 2018). Possible anti‐inflammatory roles of phycocyanin are linked with the modulation of Toll‐like Receptor (TLR) and nuclear factor‐kappa B (NF‐κB) signaling pathways (Liu et al. 2022). Beta‐carotene serves as a protective agent against singlet oxygen‐induced lipid peroxidation. It also interacts with the intracellular accumulation of reactive oxygen species (ROS), and importantly, it downregulates the expression of inflammatory genes (Deng and Chow 2010). Furthermore, GLA is converted into dihomogamma linolenic acid (DGLA), which is subsequently metabolized by cyclooxygenases and lipoxygenases to produce anti‐inflammatory eicosanoids (Kapoor and Huang 2006). The main mechanism for the anti‐inflammatory properties of tocopherols is related to the inhibition of protein kinase B (AKT) pathway (Kapoor and Huang 2006). The anti‐inflammatory activities of polyphenols included inhibition of some enzymes with proinflammatory functions such as inducible nitric oxide synthase (iNOS), lipoxygenase (LOX), and Cyclooxygenase‐2 (COX‐2) (Hussain et al. 2016). Figure 7 presents a summary of the potential anti‐inflammatory mechanisms for the main components of spirulina.

Previous studies have indicated the potential utility of Spirulina as a nutraceutical food for managing inflammation, as assessed through measurements of plasma interleukin‐2 (IL‐2) levels, adiponectin levels, and plasma concentrations of tumor necrosis factor‐α (TNF‐α) (Vázquez‐Velasco et al. 2015; Park et al. 2008). Supplementing with Spirulina has been demonstrated to help elite rugby players avoid inflammation and skeletal muscle damage caused by exercise by lowering levels of CK, CRP, and F2‐Isop both immediately after exercise and 24 h later (Milasius et al. 2009). Little is known about the mechanisms underlying the aforementioned effects of Spirulina on exercise conditions.

Also, human and laboratory research has shown that Spirulina can help reduce insulin resistance. This is due to its antioxidative and anti‐inflammatory effects, which promote insulin secretion (Fujimoto et al. 2012; Gargouri et al. 2016). Obesity is correlated with an elevation in pro‐inflammatory cytokines. However, Spirulina's immunomodulatory and anti‐inflammatory characteristics can play a role in reducing their production (Zarezadeh et al. 2021; Moradi et al. 2019). Other research suggests that Spirulina might contribute to weight loss. As a result, Spirulina could potentially alleviate obesity‐induced oxidative stress and inflammation (Yousefi et al. 2018). Furthermore, Spirulina has been associated with potential weight‐loss advantages and appetite regulation. Reducing oxidative stress and inflammation induced by obesity could constitute another way Spirulina contributes to combating inflammation (Zeinalian et al. 2017). Through its dual action as an antioxidant and an anti‐inflammatory, Spirulina blocks the inflammatory response and the oxidative stress‐inflammatory signal transduction pathways (Pak et al. 2012). On the other hand, Phycocyanin significantly decreased myeloperoxidase (MPO) activity, which had been elevated in the control group of colitis patients following 24 h (Abdel‐Daim et al. 2015). Furthermore, histopathological and ultrastructural investigations demonstrated that rats treated with phycocyanin experienced a degree of inhibition in inflammatory cell infiltration and a reduction in colonic damage (Abdel‐Daim et al. 2015).

In the current study, Spirulina supplementation significantly reduced serum CRP levels compared to the control group. Another study showed that the Spirulina group had a statistically significant 26.8% lower hs‐CRP level than the baseline. Moreover, a non‐significant 8.6% increase in adiponectin levels was observed compared to the baseline (Yousefi et al. 2018). Possible explanations for the intervention's moderating effect on hs‐CRP include the anti‐inflammatory properties of Spirulina components or a more substantial weight loss within the intervention group (Yousefi et al. 2018). Multiple studies have indicated inflammation, antioxidant activity, and oxidative stress improve after consuming Spirulina supplements (Lee et al. 2008). Two studies show that Spirulina supplementation of either 2 g or 5 g per day can increase total antioxidant activity (Szulinska et al. 2017; Winter et al. 2014). In one study, adding 2 g of Spirulina to the diets of obese and overweight people for 12 weeks led to a noticeable decrease in hs‐CRP (Yousefi et al. 2018). Various factors could potentially influence the connections between Spirulina supplementation and the biomarkers related to inflammation and oxidative stress. These factors may encompass the initial levels of inflammatory markers and oxidative stress in the individuals, their ethnic background, the quantity of Spirulina consumed, and the study duration (Deng and Chow 2010). Spirulina has been suggested as an effective supplement for combating inflammation, primarily due to its antioxidative properties. It achieves this by neutralizing reactive oxygen species and free radicals, inhibiting the activity of factor KB, and decreasing the production of proinflammatory cytokines (Riss et al. 2007).

Measuring CRP levels can aid in the early detection of individuals at risk of developing hypertension and cardiovascular disease. On the contrary, CRP might raise blood pressure and advance atherogenesis through mechanisms like inhibiting endothelial nitric oxide production (Shafi Dar et al. 2010). A study revealed that individuals with Alzheimer's disease (AD) who were administered Spirulina for 12 weeks experienced noteworthy reductions in their hs‐CRP levels. However, their NO, TAC, GSH, and MDA levels remained unaffected (Tamtaji et al. 2023).

The current meta‐analysis boasts several strengths. Based on our best knowledge, this study is the first meta‐analysis to comprehensively review the available findings on the effect of Spirulina supplementation on CRP levels in humans. Performing meta‐regression and dose–response analysis was another strength of this review. However, it is essential to acknowledge certain limitations when interpreting these findings. In some analyses, there was notable heterogeneity among the studies incorporated. Furthermore, the number of studies included in this meta‐analysis was relatively limited. Consequently, it is essential to approach the data from this study cautiously and be mindful of potential generalizations.

## 5 Conclusion

Our findings indicate a significant reduction in CRP levels followed by Spirulina supplementation in adults. As a result, it seems plausible to present Spirulina supplementation as a supplementary approach alongside primary treatments, such as medications, for individuals dealing with chronic diseases to help reduce inflammation. However, due to the high heterogeneity among the included studies and the borderline significance of the effectiveness of Spirulina supplementation on CRP levels, the results of this study may not be used with certainty. Therefore, it is suggested that confirmation of this effectiveness requires larger‐scale and meticulously structured studies to provide more comprehensive insights and substantiate the observed effects.

## Author Contributions

**Mostafa Shahraki Jazinaki:** conceptualization (equal), data curation (equal), formal analysis (equal), investigation (equal), methodology (equal), project administration (equal), writing – original draft (equal), writing – review and editing (equal). **Mohammad Rashidmayvan:** investigation (equal), methodology (equal), writing – original draft (equal). **Pegah Rahbarinejad:** investigation (equal), methodology (equal), writing – original draft (equal). **Mohammad Reza Shadmand Foumani Moghadam:** writing – review and editing (equal). **Naseh Pahlavani:** conceptualization (equal), investigation (equal), supervision (equal), writing – review and editing (equal).

## Ethics Statement

The authors have nothing to report.

## Consent

The authors have nothing to report.

## Conflicts of Interest

The authors declare no conflicts of interest.

## Data Availability

The data that support the findings of this study are available from the corresponding author upon reasonable request.
